# Retraction Note: Re-engineered BCG overexpressing cyclic di-AMP augments trained immunity and exhibits improved efficacy against bladder cancer

**DOI:** 10.1038/s41467-026-70972-5

**Published:** 2026-03-23

**Authors:** Alok Kumar Singh, Monali Praharaj, Kara A. Lombardo, Takahiro Yoshida, Andres Matoso, Alex S. Baras, Liang Zhao, Geetha Srikrishna, Joy Huang, Pankaj Prasad, Jonathan D. Powell, Max Kates, David McConkey, Drew M. Pardoll, William R. Bishai, Trinity J. Bivalacqua

**Affiliations:** 1 Johns Hopkins University, School of Medicine, Department of Medicine, Center for Tuberculosis Research, Baltimore, MD USA; 2 The Bloomberg-Kimmel Institute for Cancer Immunotherapy at Johns Hopkins, Baltimore, MD USA; 3 Johns Hopkins University, School of Medicine, Department of Urology, Baltimore, MD USA; 4 Department of Urology, Hyogo Prefectural Nishinomiya Hospital, Nishinomiya, Japan; 5 Department of Pathology, The Johns Hopkins University, Baltimore, MD USA; 6 Perelman School of Medicine at the University of Pennsylvania, Division of Urology, Department of Surgery, Philadelphia, PA USA

Retraction to: *Nature Communications* 10.1038/s41467-022-28509-z, published online 15 February 2022

The authors have retracted this article because there are duplicate cells in the Source Data for the following figure panels: Figure 1 panels d and e, Figure 2 panels a,b c and h, Figure 3 panels c and d, Figure 5 panel b, Figure 6 panels b and c, Figure 7 panels b, c and d, Figure S2 panels b, c and d, Figure S3 panel b, Figure S12, Figure S13 panels b, d and e, Figure S14 panels a, b, c and d, Figure S15 panel b, and Figure S16 panels a and b. Please note that the Source Data tab labelled Suppl. 13b actually pertains to Figure S13c.

All authors agree with this retraction.

